# Adapting Logic to Physics: The Quantum-Like Eigenlogic Program

**DOI:** 10.3390/e22020139

**Published:** 2020-01-24

**Authors:** Zeno Toffano, François Dubois

**Affiliations:** 1 Université Paris-Saclay, CNRS, CentraleSupélec, Laboratoire des signaux et systèmes, 91190 Gif-sur-Yvette, France; 2 Conservatoire National des Arts et Métiers, AFSCET, 75003 Paris, France; francois.dubois@u-psud.fr

**Keywords:** probabilistic logic, quantum computing gates, operator algebra

## Abstract

Considering links between logic and physics is important because of the fast development of quantum information technologies in our everyday life. This paper discusses a new method in logic inspired from quantum theory using operators, named Eigenlogic. It expresses logical propositions using linear algebra. Logical functions are represented by operators and logical truth tables correspond to the eigenvalue structure. It extends the possibilities of classical logic by changing the semantics from the Boolean binary alphabet {0,1} using projection operators to the binary alphabet {+1,−1} employing reversible involution operators. Also, many-valued logical operators are synthesized, for whatever alphabet, using operator methods based on Lagrange interpolation and on the Cayley–Hamilton theorem. Considering a superposition of logical input states one gets a fuzzy logic representation where the fuzzy membership function is the quantum probability given by the Born rule. Historical parallels from Boole, Post, Poincaré and Combinatory Logic are presented in relation to probability theory, non-commutative quaternion algebra and Turing machines. An extension to first order logic is proposed inspired by Grover’s algorithm. Eigenlogic is essentially a logic of operators and its truth-table logical semantics is provided by the eigenvalue structure which is shown to be related to the universality of logical quantum gates, a fundamental role being played by non-commutativity and entanglement.

## 1. Introduction and Motivation

One of the main goals of this research is to look for links between logic and quantum operator algebra in Hilbert space that could lead to new developments in the field of quantum information and quantum computation.

It is widely accepted that there is a difficulty in explaining quantum effects by the means of propositional logic due to the peculiar features of quantum mechanics such as interference effects and the non-commutativity of operators. An example is the frequently quoted example of the *double slit experiment*, whose protocol can be separated into three elementary propositions A, B and C:

- prop. A: “the electron is detected at position *x* ”
- prop. B: “the electron went through slit 1”
- prop. C: “the electron went through slit 2”

The measurement outcome of the experiment shows that, when combining these propositions, they do not verify the distributive law of classical logic given by:

(1) A∧(B∨C)=(A∧B)∨(A∧C)

This is because the interference effect between the two slits, which is a purely quantum effect for particles, represented by the logical disjunction function B∨C, cannot be physically separated into two possible events represented respectively by the two conjunctions A∧B and A∧C.

This fact has motivated the research for more quantum-adapted logical systems. John von Neumann proposed a correspondence between projection operators and logical propositions in his 1932 book [1]. Propositions of a Boolean algebra satisfying the distributive law (1) can be represented only by commuting projection operators i.e., compatible operators in quantum mechanics.

The successive work in logic by Birkhoff and von Neumann [2] substituted Boolean algebras with the lattice of closed subspaces of a (finite) Hilbert space. The method was successively named *Quantum Logic*. It evolved into an independent discipline with many followers but also critics. Despite many advances, this discipline is still not considered as an operational tool for quantum computing.

A controversial argument against quantum logic was given by the French logician Jean-Yves Girard in [3]: “*To imagine foundations, if not «quantum», at least in a quantum spirit: proportionately speaking, something of the sort Alain Connes is doing with non-commutative geometry. That is the project of the day, enough to be kept busy for a while! Which topsy-turvies the usual relation logic/quantum: instead of interpreting quantum in logic, one tries the opposite*”.

An improvement of quantum logic has been proposed, inspired by quantum computing research, leading to the quantum computational logic approach [4] where any language formula in logic can be considered as a compact logical description of a quantum circuit.

The work presented here is inspired from George Boole’s elective expansion method in logic [5] and presents an operational and geometric approach to logic named Eigenlogic [6]. This method shows that Boole’s symbolic arithmetic formulation can naturally be translated into the language of linear algebra in terms of operators. In this way, propositional logic can be represented by means of combinations of elementary operators linked by the Kronecker tensor product.

A fact that came out from this research is that there exist better adapted logical mathematical formulations using alternative alphabets than the usual binary alphabet using the Boolean numbers 0 and 1. Paul Benioff, one of the pioneers of quantum Turing machines, seems to go in this direction identifying different number systems adapted to specific quantum system such as angular momentum, harmonic oscillator, canonical positon and momentum continuous variables. [7].

There is an increasing interest in applying many-valued logic to the explanation of quantum phenomena and this is also linked with the rapidly growing field of fuzzy set theory [8]. It also has to be reminded that the mathematical theory of quantum physics and the theory of many-valued logic, whose founders are Jan Łukasiewicz [9] and Emil Post [10], were created nearly simultaneously in the second and third decade of the twentieth century.

Many-valued logic was thoroughly investigated in the years 1970–1980 proposing new circuit designs for all possible logical and computing operations [11]. But few practical industrial applications emerged, because the modern digital semiconductor technology, based on logical switching theory, has almost exclusively adopted binary solutions. But there is a serious hope that various future quantum technologies will integrate many-valued circuits using *qudits* because of the advantage brought by more compact solutions than the binary ones. It has to be stressed that multi-level systems (having a state-space dimension greater than two) are ubiquitous in quantum physics, an important example is *quantum angular momentum* where the binary *spin ½* system is only a particular case.

A consistent logical program should also include first order predicate logic and must be able to tackle logical problems of incompleteness, non-computability i.e., Turing machines. It will be conjectured here, in the context of Eigenlogic, that operator non-commutativity and entanglement make an interesting incursion in this direction, by the means of a new interpretation of the logical syntactic-semantic duality and of quantum-gate logical universality.

The paper is organized as follows:

Section 2 describes the general Eigenlogic method for a binary system using projection operators in the alphabet {0,1} and then for a binary system using involution operators in the alphabet {+1,−1}. The correspondence with standard quantum computing gates is also discussed. The section closes with the general operator interpolation method for whatever alphabet in many-valued logic.

Section 3 addresses the problem of the measurement of logical operators outside their logical eigensystem. It starts with the description of the probability interpretation of Boole and Poincaré based on logic showing parallels with the successive discussion on fuzzy Eigenlogic.

Section 4 presents logical formulations using operators. Starting with Von Neumann’s “projections as propositions” quantum logic and then presenting the less-known logical interpretation of quaternions by George Boole leading to a natural quantum interpretation because of the isomorphism between quaternions and Pauli matrices. The generalized Pauli operators of the Weyl–Heisenberg group are also analyzed for their connection with logical semantic-syntactic duality and many-valued logic.

Section 5 proposes several conjectures indicating new methods using logical operators for universality of logical quantum gates, first order logic, Post normal systems and Combinatory logic.

## 2. Eigenlogic

Eigenlogic [6] stems from a simple idea that wants to transcribe propositional logic in a matrix linear algebra context.

This view makes the following correspondence between linear algebra and logic:
eigenvalues↔logical truth valuesoperators↔logical connectiveseigenvectors↔logical interpretations

In Eigenlogic, logical operators can be matched to every logical function (a logical connective). The values in logical truth tables correspond to the logical operator’s eigenvalues. With this method, propositional logic can be expressed using elementary quantum operators combined by the Kronecker tensor product. The result of a “measurement” on an operator will give the truth value of the associated logical proposition. This measurement is logically interpretable when applied to vectors in the eigenvector space, leading to an original view of the measurement postulate in quantum physics.

### 2.1. Boolean Eigenlogic Using Zero and One

George Boole used mathematical symbols taking the values 0 and 1 representing *resp.* the “False” or “True” value of a proposition [5]. He used an *idempotent* symbol x (named *elective symbol*) obeying the equation: $x^{2}=x$ with only two possible solutions: 0 and 1. This equation was considered by Boole the “fundamental law of thought” [12]. In Eigenlogic, the algebra of idempotent elective symbols can be represented by a set of commuting projection operators [6].

Using the elective decomposition of binary logical connectives as originally introduced by George Boole, the expression of a logical operator for one input (*arity*-1) is directly written in the form of a spectral decomposition:

(2) $$F_{1}=f\left(0\right)\Pi_{0}+f\left(1\right)\Pi_{1}=diag\left[f\left(0\right),f\left(1\right)\right]$$

The operators used in (2) are 2-dimensional rank-1 projectors $\Pi_{0}=I_{2}-\Pi$ and $\Pi_{1}=\Pi$, where the operator Π is in Eigenlogic the *seed operator*. The *cofactors* f(0) and f(1) are the eigenvalues of the operator $F_{1}$ taking the values 0 and 1. $F_{1}$ is also an idempotent projection operator. Logical negation corresponds here to complementation and is obtained by subtracting the negated operator from the identity operator I. The two important properties are thus:

(3) $$\mathrm{idempotence}:\;F^{2}=F,\;\mathrm{complementation}:\;\bar{F}=I-F$$

The eigenvectors of $F_{1}$, named |0〉 and |1〉, form the canonical basis for one *qubit*, and correspond also to the spin ½ north and south poles on the unitary Bloch sphere.

For two inputs (arity-2) the logical operators $F_{2}$ are obtained by using the Kronecker product ⊗ and the seed operator Π. The four compound projectors correspond to the four quantum state density matrices for 2 qubits:

(4) $$\Pi_{00}=|00\rangle \langle 00|\;,\;\Pi_{01}=|01\rangle \langle 01|\;,\;\Pi_{10}=|10\rangle \langle 10|\;,\;\Pi_{11}=|11\rangle \langle 11|=\Pi ⊗\Pi$$

The multilinear expansion using truth values f(x,y) for two inputs x,y ∈{0,1} is then:

(5) $$F_{2}=f\left(0,0\right)\Pi_{00}+f\left(0,1\right)\Pi_{01}+f\left(1,0\right)\Pi_{10}+f\left(1,1\right)\Pi_{11}$$

In propositional logic, every logical function can be expressed as a function of its inputs. Here the two inputs are given by two *logical projectors* corresponding to the operators A and B:

(6) $$A=\Pi ⊗I_{2}=\mathrm{diag}\left(0,0,1,1\right)\;,\;B=I_{2}⊗\Pi =\mathrm{diag}\left(0,1,0,1\right)$$

The extensions in (6) of the seed operator Π by the identity operator $I_{2}$ using the Kronecker product ensures the *independence* of A and B which are considered in Eigenlogic the *atomic propositions*. This is a major difference with traditional quantum logic where atomic propositions are pure quantum state density matrices i.e., rays (rank-1 projection operators).

All the other logical operators (see Table 1) can then be obtained directly from A and B as is usual in propositional logic.

The case n=1 provides four logical operators $F_{1}$ obtained from (2): projection $\Pi_{1}=\Pi$, negation $\Pi_{0}=I_{2}-\Pi$, tautology $I_{2}$ (the identity matrix) and contradiction $0_{2}$ (the zero matrix).

For n=2 one has 16 binary logical operators $F_{2}$ from (5). These are uniquely characterized by their truth table given in Table 1 with their associated projection operators.

In general, the total number of logical binary connectives is $2^{2^{n}}$ for an arity-n system. For an m-valued alphabet (m>2 corresponding to many-valued logic) and an arity-n logical system the number of logical connectives becomes $m^{m^{n}}$ [10,11].

### 2.2. Involution Eigenlogic Using the Numbers +1 and −1

There exists an isomorphism between a projection operator F and an involution operator G given by the Householder Transform:

(7) $$G=I-2F={\left(-1\right)}^{F}=e^{i\pi F}=e^{i\frac{\pi }{2}}e^{-i\frac{\pi }{2}G}$$

The eigenvalues of G, the correspondent to the eigenvalues 0 and 1 of F representing “False” and “True”, are +1=1−2⋅0 and −1=1−2⋅1 using (7). So the alphabet of this logical binary system is {+1,−1}. The operators F and G commute with equal degeneracy and thus share the same eigenvectors. The seed operator for this system is the Pauli matrix ${\hat{\sigma }}_{z}$ (the Z gate):

(8) $${\hat{\sigma }}_{z}=Z=I_{2}-2\Pi =\left(\begin{array}{cc} +1 & 0\\ 0 & -1 \end{array}\right)$$

Hereafter the eigenbasis of ${\hat{\sigma }}_{z}$ is chosen as the reference basis and corresponds to the usual qubit *computational basis* used in quantum circuits. The choice of ${\hat{\sigma }}_{z}$ is conventional and one could have chosen another Pauli matrix as the seed operator. In Eigenlogic, every basis choice generates a new logical system, this will be discussed later in relation to the logical semantic interpretation.

For arity-2, in the {+1,−1} system , *logical dictators* [13] U and V, the equivalent of the *logical projectors* A and B for the system {0,1}, are:

(9) $$U=Z⊗I_{2}=diag\left(+1,+1,-1,-1\right),\;V=I_{2}\;⊗Z=diag\left(+1,-1,+1,-1\right)$$

All these operators are involutions and the logical negation in the {+1,−1} system is obtained by multiplying by −1:

(10) $$\mathrm{involution}:\;G^{2}=I,\;\mathrm{negation}:\;\bar{G}=-G$$

The formulation using these involution operators G gives the same results as the *Quantum Boolean Functions* approach proposed in [13] based on the mathematical method of *Fourier transform of Boolean functions*.

Binary logic using {+1,−1} appears more adapted to certain quantum systems because it includes a negative number. For example the spin ½ system with the positive and negative eigenvalues ½ℏ and −½ℏ is proportional to the alphabet {+1,−1}.

Even if the logical truth-table structure is equivalent for the two alphabets {0,1} and {+1,−1}, the mathematical expressions and operations for a given logical function are not the same. A simple and very important example is exclusive disjunction XOR which becomes a product in the alphabet {+1,−1} whereas the product in the alphabet {0,1} corresponds to the conjunction AND. This is in our view a very important technical fact.

All the 16 logical involution operators $G_{2}$ for the alphabet {+1,−1} are given on Table 1.

It has to be outlined that the binary alphabet {+1,−1} is currently used in *spin-glass* and *Ising* computer models, where +1 (False) is spin up and −1 (True) spin down.

### 2.3. Eigenlogic Operators and Quantum Computing Gates

Quantum gate optimization in quantum circuits is a strategic issue for quantum computing and quantum simulation. Quantum reversible gates representing Boolean functions have been widely analyzed by different implementation methods using for example the 2-qubit CONTROL-NOT and the 3-qubit Toffoli gates or the *non-Clifford gates* such as the T-gate: $T=Z^{1/4}$. Essentially all the proposed methods are based on the conditional quantum logic paradigm originally proposed by David Deutsch in [14]. The CONTROL-NOT gate on two qubits being its basic element (see $C_{\mathrm{NOT}}$ given in (14)). The operation on a 2-qubit state is |x,y〉→|x,x⊕y〉. The exclusive disjunction (XOR,⊕) negates the target qubit y when the control qubit x is one and leaves it unchanged when x is zero. The logical gate for negation (NOT) is the Pauli ${\hat{\sigma }}_{x}$ operator (see (37)) named the X-gate.

A universal quantum logic gate is the 3-qubit doubly-CONTROL-NOT gate (Toffoli gate) with the logical operation |x,y,z〉→|x,y,(x∧y)⊕z〉 that negates the target bit z when the conjunction x∧y on the two control qubits x and y is satisfied (both must be 1). This gate is equivalent to a negated binary conjunction NAND logical gate, which is known to be universal in classical propositional logic. These quantum gates transform the qubits as reversible permutation operators and are thus non-diagonal in the computational basis.

A quantum gate which is diagonal in the computational basis is the CONTROL-Z gate, named here $C_{Z}$. When the control bit is at one it implements the Pauli ${\hat{\sigma }}_{z}$ operator, the Z-gate of (8). Concretely this means that the output is multiplied by −1 when the control and target bits are both at one and is unchanged otherwise. So by looking at the eigenvalues of $C_{Z}$ one sees that this operator corresponds to the Eigenlogic conjunction operator $G_{A\wedge B}=G_{\mathrm{AND}}$ (AND, ∧). Its expression is obtained directly using the dictators U and V of (9) giving the known expression [15,16]:

(11) $$C_{Z}=\mathrm{diag}\left(1,\;1,\;1,-1\right)=\frac{1}{2}\left(I_{4}+U+V-U\cdot V\right)$$

One can also derive this operator using the Householder transform (7) of the projection logical operator $F_{\mathrm{AND}}$.

(12) $$\begin{array}{c} G_{\mathrm{AND}}=I_{4}-2F_{\mathrm{AND}}={\left(-1\right)}^{\Pi ⊗\Pi }=\mathrm{diag}\left(1,\;1,\;1,-1\right)=C_{Z}\\ G_{\mathrm{AND}}|xy\rangle \;=C_{Z}\;|xy\rangle \;={\left(-1\right)}^{xy}\;|xy\rangle \end{array}$$

Applying the operator $C_{Z}$ on the state |xy〉 is equivalent to multiplying by ${\left(-1\right)}^{xy}$, and the value −1, for xy=1*,* is only obtained when the input state is |11〉.

The expression of the CONTROL-NOT operator, named here $C_{\mathrm{NOT}}$, can also be obtained straightforwardly, using for the control qubit the seed projection operator Π=|1〉〈1| of the computational basis (the ${\hat{\sigma }}_{z}=Z$ eigenbasis) and for the target qubit the seed projection operator $\Pi_{X}=|-\rangle \langle -|$ of the eigenbasis of X, where the eigenstate is $|-\rangle =\frac{1}{\sqrt{2}}\left(|0\rangle -|1\rangle \right)$. The projection operator $\Pi_{X}$ is derived using the Hadamard gate H [15]. These operators are:

(13) $$\Pi =|1\rangle \langle 1|\;=\left(\begin{array}{cc} 0 & 0\\ 0 & 1 \end{array}\right)\;,\;H=\frac{1}{\sqrt{2}}\left(\begin{array}{cc} 1 & 1\\ 1 & -1 \end{array}\right)\;,\;\Pi_{X}=|-\rangle \langle -|=H\cdot \Pi \cdot H=\frac{1}{2}\left(\begin{array}{cc} 1 & -1\\ -1 & 1 \end{array}\right)$$

Using the analogy with the $C_{Z}$ gate, one defines the projection operator $\Pi_{C_{\mathrm{NOT}}}$, associated to the $C_{\mathrm{NOT}}$ gate, giving the following form and the corresponding matrix representation:

(14) $$\Pi_{C_{\mathrm{NOT}}}=\Pi ⊗\Pi_{X}\;,\;C_{\mathrm{NOT}}=I_{4}-2\Pi_{C_{\mathrm{NOT}}}={\left(-1\right)}^{\Pi ⊗\Pi_{X}}=\left(\begin{array}{c} \begin{array}{cc} 1 & 0\\ 0 & 1 \end{array}\;\begin{array}{cc} 0 & 0\\ 0 & 0 \end{array}\\ \begin{array}{cc} 0 & 0\\ 0 & 0 \end{array}\;\begin{array}{cc} 0 & 1\\ 1 & 0 \end{array} \end{array}\right)$$

The same method can be applied to build the Toffoli gate (doubly-CONTROL-NOT) named here TO. One starts with with a 3 qubit Eigenlogic conjunction $G_{A\wedge B\wedge C}=I_{8}-2\left(\Pi ⊗\Pi ⊗\Pi \right)$ and Hadamard transforms the last qubit. The polynomial expression is then easily calculated:

(15) $$TO\;=I_{8}-2\left(\Pi ⊗\Pi ⊗\Pi_{X}\right)=\frac{1}{2}\left(I_{8}+Z⊗I_{4}+I_{2}⊗C_{\mathrm{NOT}}-Z⊗C_{\mathrm{NOT}}\right)$$

In the design of quantum circuits one never considers directly addition of quantum gates, as the operator expressions given in (11) and (15), one prefers expressions using products of quantum gates because of the reversible unitary transfer structure of the circuit. There is a procedure to transform a sum into a product using the Householder transform (7). For example the $C_{Z}$ gate polynomial expression can be transformed by (7) into a product of unitary exponentials of operators:

(16) $$C_{Z}\;=e^{i\frac{\pi }{2}}e^{-i\frac{\pi }{2}C_{Z}}=e^{i\frac{\pi }{2}}e^{-i\frac{\pi }{4}\;\left(I+U+V-U\cdot V\right)}=\;e^{i\frac{\pi }{4}}\;e^{-i\frac{\pi }{4}\;U}\;e^{-i\frac{\pi }{4}\;V}\;e^{+i\frac{\pi }{4}\;U\cdot V}$$

The product factorization is possible because, belonging to the same Eigenlogic family, all the operators in the exponential argument commute and the order of the multiplication can be interchanged. A similar method can be used for the CONTROL-NOT $C_{\mathrm{NOT}}$ and Toffoli TO gates [16]. For a more detailed discussion of the method and other examples of these methods see [16].

### 2.4. Operators for Many-Valued Logic Using Lagrange Interpolation and the Cayley–Hamilton Theorem

The Cayley–Hamilton theorem is a very powerful theorem in linear algebra and has an important consequence, as stated by the mathematician Edward Fromanek in [17]: “*all polynomial identities on* n × n *complex matrices are consequences of the CayIey-Hamilton theorem*…”. The theorem says that any finite matrix is the solution of its own characteristic equation.

In Eigenlogic, this theorem is used to generalize the mathematical framework of propositional logic to values different from the Booleans {0,1}. The method is based on the classical *Lagrange interpolation* method where the “variable” is replaced by an Eigenlogic non-degenerate seed operator with m distinct eigenvalues. As will be shown hereafter this method permits to determine unique logical operators for whatever numerical values. In the many-valued logic case, popular choices are, the natural numbers {0,1,2,…,m} formalized in Post’s logic [10] and the rational fractional numbers in the unit interval [0,1], the set $\left\{0,\frac{1}{m},\frac{2}{m},\ldots ,\frac{m-1}{m},1\right\}$ of m+1 values, in Łukasiewicz’s logic [9]. Other numerical choices can include negative numbers, this is the case, for example, of the balanced ternary system {+1,0,−1} used for *qutrits* discussed hereafter.

Complex numbers can also be considered as logical values. For example, those adapted to the *Quantum Fourier Transform* using the roots of unity giving for a logical system of dimension m the set $\left\{e^{i2\pi 0},e^{i2\pi \frac{1}{m}},\;e^{i2\pi \frac{2}{m}},\ldots ,e^{i2\pi \frac{m-1}{m}}\right\}$. These values are also the eigenvalues of the generalized Pauli or Weyl–Heisenberg operators $Z_{m}$ and $X_{m}$ given in (41,42) that will be analyzed in Section 4.3.

In general one starts by defining the seed operator Λ with m non-degenerate eigenvalues $\lambda_{i}$. The eigenstate density matrices, $\left|\lambda_{i}\rangle \langle \lambda_{i}\right|$ which are rank-1 projection operators $\Pi_{\lambda_{i}}$, are calculated for each eigenvalue. Lagrange interpolation directly gives the density matrices:

(17) $$\Pi_{\lambda_{i}}\left(\Lambda \right)=\left|\lambda_{i}\rangle \langle \lambda_{i}\right|=\overset{m}{\underset{j=1,j\neq i}{\prod }}\frac{\Lambda -\lambda_{j}I_{m}}{\lambda_{i}-\lambda_{j}}$$

Cayley–Hamilton’s theorem shows that this development is unique. The formal expression is a polynomial in Λ up to the power m−1. The operator is represented by a m×m square matrix.

In logic, in order to get interpretable propositions, both functions and arguments must take the same values. This signifies that an arity-1 logical function ℓ(λ) having its domain on the set of *m* distinct logical values $\lambda_{i}$, takes also its values in this set: $\ell \left(\lambda_{p}\right)\in \left\{\lambda_{1},\lambda_{2},.,\lambda_{p},\ldots ,\lambda_{m}\right\}$. The corresponding logical operator spectral decomposition is then directly obtained by interpolation:

(18) $$F_{\ell }=\overset{m}{\underset{i=1}{\sum }}\ell \left(\lambda_{i}\right)\;\Pi_{\lambda_{i}}$$

The quantum *observable* (i.e., Hermitian operator) *orbital angular momentum* is defined by two quantum numbers: ℓ and $m_{l}$. ℓ is an integer ≥0 and $m_{l}$ obeys $-\ell \leq m_{l}\leq \ell$ by steps of 1. $\hbar m_{l}$, is the eigenvalue of the *z*-component of the orbital angular momentum observable $L_{z}$. The matrix of this operator for ℓ=1 is:

(19) $$L_{z}=\hbar \Lambda =\hbar \left(\begin{array}{ccc} +1 & 0 & 0\\ 0 & 0 & 0\\ 0 & 0 & -1 \end{array}\right)$$

Using the operator Λ of (19) as the Eigenlogc seed operator, one can calculate the balanced ternary logic operators. These operators are explicitly derived from the interpolation formula (18) using the rank-1 projection operators $\Pi_{+1}$, $\Pi_{0}$ and $\Pi_{-1}$ for the three eigenstates |+1〉, |0〉 and |−1〉 of $L_{z}$, defining a *qutrit*. All logical operators are combinations of these projection operators_,_ their expression as a function of the seed operator Λ are calculated using (17):

(20) $$\Pi_{+1}=\frac{1}{2}\Lambda \left(\Lambda +I_{3}\right)\;,\;\Pi_{0}=I_{3}-\Lambda^{2}\;,\;\Pi_{-1}\;=\frac{1}{2}\Lambda \left(\Lambda -I_{3}\right)$$

All arity-1 logical operators $F_{\ell }\left(\Lambda \right)$ can then be derived using (18).

In the case of an arity-2 system, the logical operators are represented by 9×9 matrices. The dictators, U and V, are then defined in the same way as in (9):

(21) $$U=\Lambda ⊗I_{3}\;,\;V=I_{3}⊗\Lambda \;,\;U\cdot V=\Lambda ⊗\Lambda$$

In many-valued logic, the universal connectives Min and Max [11] are the equivalent of the binary AND and OR. The Eigenlogic operators for a balanced ternary qutrit system are [6,16]:

(22) $$Min=\frac{1}{2}\left(U+V+U^{2}+V^{2}-U\cdot V-U^{2}\cdot V^{2}\right)=\mathrm{diag}\left(+1,+1,+1,+1,0,0,+1,0,-1\right)$$

(23) $$Max=\frac{1}{2}\left(U+V-U^{2}-V^{2}+U\cdot V+U^{2}\cdot V^{2}\right)=\mathrm{diag}\left(+1,0,-1,0,0,-1,-1,-1,-1\right)$$

## 3. Probabilities and Fuzzy Eigenlogic

### 3.1. Probability Theory: The Views of Boole and Poincaré

The work of George Boole included also the analysis of hypothetical propositions, which led him to define probabilities directly from logic [12]. The algebraic method on symbols representing classes used by Boole, was formalized successively into the theory of *Boolean algebra*. It has to be emphasized that probability theory applied to discrete sample spaces, is defined over subsets using the operations of intersection, union and complementation characteristic of a Boolean algebra.

A similar approach to Boole’s interpretation of probabilities was given successively by Henri Poincaré in [18] in which he made a simple and natural analysis of probabilities by classifying them for all possible events associated to the withdrawal experiment of two objects, A and B, from an urn.

Considering the combination of events A and B, different probabilities can be defined, depending on whether any of these events occur, or both, or neither. In this way, as is done in logic for conjunction and negation, one defines the four compound events where AB represents the event where both A and B occur, $A\bar{B}$ where A occurs and B does not occur, $\bar{A}B$ where A does not occur and B occurs, and $\bar{A}\bar{B}$ where neither events occur. In an actual experiment one counts the number of occurrences of these different events, for example for the first compound event AB we will count the number $n_{\mathrm{AB}}$. Probabilities will be considered as the ratio between the number corresponding to the considered event divided by the total number of events. Then all possible probabilities are a function of only four numbers $n_{\mathrm{AB}}$, $n_{A\bar{B}}$, $n_{\bar{A}B}$, and $n_{\bar{A}\bar{B}}$ considered as a basis. Their sum being the total number of events n given by the sum $n=n_{\mathrm{AB}}+n_{A\bar{B}}+n_{\bar{A}B}+n_{\bar{A}\bar{B}}$.

For Poincaré probability, calculus is based on two theorems: the theorem of total probability and the theorem of composed probabilities.

The probability of event AB corresponds to a logical conjunction AND (∧) event. The probability for the disjunction event OR (∨), where one at least of the two events occurs, is then obtained straightforwardly. The expressions for the different event probabilities are:

(24) $$p_{A\wedge B}=\frac{n_{\mathrm{AB}}}{n}\;,\;p_{A\vee B}=\frac{n_{\mathrm{AB}}+n_{A\bar{B}}+n_{\bar{A}B}}{n}\;,\;p_{A}=\frac{n_{\mathrm{AB}}+n_{A\bar{B}}}{n}\;,\;p_{B}=\frac{n_{\mathrm{AB}}+n_{\bar{A}B}}{n}$$

when combined, these probabilities lead to the following general expressions:

(25) $$p_{A\wedge B}+p_{A\vee B}=p_{A}+p_{B}\;\mathrm{or}\;p_{A\vee B}=p_{A}+p_{B}-p_{A\wedge B}$$

The above expression (25) is the simplest form of the renowned *inclusion-exclusion rule* (form on the right in (25)) also named the *Boole–Sylvester rule* in the English community or the *formule du crible de Poincaré* in the French community. In this way the theorem of total probability expressing the probability p that, at least, one out of N events occurs is a function of the combined event probabilities:

(26) $$p=\overset{N}{\underset{i=1}{\sum }}p_{i}-\overset{N}{\underset{i,j|i<j}{\sum }}p_{ij}+\overset{N}{\underset{i,j,k|i<j<k}{\sum }}p_{ijk}-\ldots$$

where the condensed notation $p_{ij}$ is used for $p_{i\wedge j}$.

In standard probability theory one generally considers only mutually exclusive events i.e., $p_{A\wedge B}=0$, in this case disjunction is equivalent to exclusive disjunction and the total probability is the sum of the individual event probabilities.

The results of the preceding discussion show that probabilities can be derived from logical propositions in a simple way, the logical structure is apparent in the expressions of the probabilities. In the next section it will be shown that the Eigenlogic fuzzy membership functions have essentially the same structure as the preceding probabilities.

### 3.2. Fuzzy Eigenlogic and Quantum Probabilities

In this Section we will show that when considering logical inputs represented by quantum states not belonging to the Eigenlogic operator eigensystem one obtains a fuzzy logic representation. *Fuzzy membership functions* characterize the domain of fuzzy sets. In Eigenlogic the fuzzy membership function corresponds to the quantum mean value (*Born rule*) of the considered logical projection operator on an input state. Fuzzy logic [8] admits truth values taking values between 0 and 1. So in some way the fuzzy logical character arises because of a *superposition* of logical propositions, the logical value of a proposition being a continuous value ranging from 0 (False) to 1 (True). This gives also an original insight to the quantum measurement postulate by the means of fuzzy logic.

In Eigenlogic, the mean value of a logical operator will provide the truth value of the associated logical proposition. The *interpretable* case, corresponding to sharp truth values, is obtained for input states belonging to the logical eigenspace associated to the Eigenlogic operator.

One can, for example, choose for the logical eigenspace the 2-qubit computational basis of the logical projection family defined in Section 2.1. A general quantum state is then expressed by a linear combination over this basis:

(27) $$|\psi \rangle =c_{00}|00\rangle +c_{01}|01\rangle +c_{10}|10\rangle +c_{11}|11\rangle$$

When more than one coefficient in (27) is non-zero one is in a fuzzy logic situation. Intuitively speaking the superposition principle of quantum states considered as logical entities is analogous to a fuzzy logic formulation using fuzzy sets where a logical entity can belong to more than one set.

For the Eigenlogic projection operator F measured on a quantum state |ψ〉, the mean value leads directly to a probability measure by the Born rule:

(28) $$p_{|\psi \rangle }=\langle \psi |F|\psi \rangle =Tr(\rho \cdot F)\;\mathrm{with}\;\rho =|\psi \rangle \langle \psi |\;\mathrm{the}\;\mathrm{density}\;\mathrm{matrix}$$

The arity-1 membership function μ(A) is obtained by the quantum mean value of the logical projector operator Π over an arbitrary 2-dimensional quantum state |ϕ〉:

(29) $$|\phi \rangle =\sin\alpha |0\rangle +\;e^{i\beta }\cos\alpha \;|1\rangle \;,\;\mu \left(A\right)=\langle \phi |\Pi |\phi \rangle =\cos^{2}\alpha =p$$

A quantum composite state |ψ〉 is built by taking the tensor Kronecker product of two individual states $|\;\phi_{a}\rangle$ and $|\;\phi_{b}\rangle$ by $|\psi \rangle =|\;\phi_{a}\rangle \;⊗\;|\;\phi_{b}\rangle$.

The fuzzy membership functions corresponding to the 2-qubit logical projectors A and B (see (6) and Table 1) measured on |ψ〉 are then given by:

(30) $$\mu \left(A\right)=\langle \psi \left|\Pi \;⊗\;I_{2}\right|\psi \rangle =p\left(1-q\right)+p\cdot q=p\;,\;\mu \left(B\right)=\langle \;\psi |I_{2}\;⊗\Pi |\psi \rangle =q$$

This shows also explicitly that the membership functions correspond to quantum probabilities.

Now let us calculate for example the conjunction AND (∧) and the disjunction OR (∨) membership functions using the operators given in Table 1, which gives:

(31) μ(A∧B)=〈ψ |A·B|ψ〉=〈ψ |Π⊗Π|ψ〉=p⋅q , μ(A∨B)= p+q−p⋅q

One observes that the fuzzy disjunction membership function μ(A∨B) is an inclusion-exclusion like expression as in (26). The quantum probabilities given by the Born rule are the fuzzy Eignelogic membership functions and have their correspondence with the probability interpretation of Boole and Poincaré.

The fuzzy membership function corresponding to the logical *material implication* (A⇒B) can be used for decision-making problems. Using the Eigenlogic operator, given in Table 1, one has:

(32) $$\mu \left(A\Rightarrow B\right)=\langle \psi \;\left|\left(I_{4}-\Pi ⊗I_{2}+\Pi ⊗\Pi \right)\right|\psi \rangle =1-p+p\cdot q$$

This function was applied to the concept of *quantum robot* introduced by Paul Benioff [19] as a first approach for describing a quantum mechanical system aware of the environment and capable of making decisions. The quantum robot model was realized by Braitenberg vehicles with a quantum logic control with input vision fuzzy stimuli [20,21]. These robots display new non-classical emergent behaviours linked to quantum-like effects and reflect contextuality due to input-state superposition and entanglement of its logical control structure.

The geometrical space corresponding to a one input (arity-1) fuzzy membership function, as considered in (29), is the Bloch sphere. This can be easily understood by the normalization condition on the coefficients of the quantum state |ϕ〉 giving the equation $\sin\alpha^{2}+\cos\alpha^{2}=1$. For compound quantum-state fuzzy membership functions the geometry becomes more complex. In this case one could have some peculiar effects when considering entangled input states [21].

Fuzzy Eigenlogic could more generally benefit the emerging field of *quantum cognition* based on the *quantum-like paradigm* which applies the mathematical quantum theory to model cognitive phenomena such as information processing by the human brain, language, decision making, human memory… [22]. One of the motivations would be that in Eigenlogc the fuzzy membership functions become quantum probabilities and, for example, the fuzzy Eigenlogic implication function (32) gives a probability measure linked to a quantum operator used in decision-making problems.

## 4. Operators in Logic: Projectors, Quaternions, Pauli Matrices and the Heisenberg Group

Logic using operators has a long history. We will recall here some of the approaches that will permit us to make links with Eigenlogic. An important difference between operators and functions is that an operator can be described by its action only, without defining the input domain for which this operation yields the required outcomes. Another important difference is that some operators do not have a strictly defined domain; they can admit any inputs, including themselves.

### 4.1. Von Neumann’s ‘Projections as Propositions’

John von Neumann noticed that the projection operators P in Hilbert space, verifying the condition $P^{2}=P=P^{†}$ (idempotence and hermiticity), can represent logical propositions by identifying the eigenvalues 0 and 1 of these operators with the truth values of the propositions. In his 1932 book [1] he states that the mathematical operations of multiplication, addition and subtraction preserving the projection properties must satisfy the following axioms:

- $P_{1}\cdot P_{2}$ is a projection operator *iff* $P_{1}\cdot P_{2}=P_{2}\cdot P_{1}$ (they commute)
- $P_{1}+P_{2}$ is a projection operator *iff* $P_{1}\cdot P_{2}=0$ or $P_{2}\cdot P_{1}=0$
- $P_{1}-P_{2}$ is a projection operator *iff* $P_{1}\cdot P_{2}=P_{2}$ or $P_{2}\cdot P_{1}=P_{2}$

This means that the idempotence property is conserved when projection operators commute, this condition is usually expressed in quantum mechanics by $\left[P_{1}\cdot P_{2}\right]=P_{1}\cdot P_{2}-P_{2}\cdot P_{1}=0$. Addition is only defined for disjoint subspaces, $P_{1}\cap^{\;}P_{2}=0$ and ordered subtraction when one subspace is included in the other $P_{2}\subseteq P_{1}$. These properties are at the origin of the motivation in Eigenlogic [6], because they establish the connection between eigenvalues and logic.

It is interesting to note that a pure quantum state |ψ〉 can also be represented by a density matrix ρ=|ψ〉〈ψ|, introduced by John von Neumann in [1]. This operator is a *ray*, a rank-1 idempotent projection operator. All these concepts lay at the foundations of quantum theory.

### 4.2. Quaternions and Their Logical Interpretation by George Boole

In a short note [23], just one year after his invention of mathematical logic in 1847 [5] George Boole, following Cayley’s interpretation of quaternions as operators, gives a logical interpretation of unitary quaternions. George Boole starts the note by giving his motivation: “*It were much to be desired that the general principles which govern the use of signs, as instruments of reasoning, were reduced to a consistent theory; for there undoubtedly exists a theory of signs applicable as well to the signs of common discourse as to the signs of mathematics*”. Then he states: “*Signs employed as instruments of reasoning may, in one point of view, be considered as the representatives of operations*”.

Considering A and B representing two given operations, the sequence of these two operations A·B will also represent an operation of the same kind if the rules of logical interpretation adopted for A and B are well defined. George Boole infers a logical interpretation of a quaternion q with a unitary constraint, the *unitary quaternion* defined by:

(33) $$q=w+ix+jy+kz\;\mathrm{with}\;w^{2}+x^{2}+y^{2}+z^{2}=1$$

The quaternion basis i, j and k verifies the following rules:

(34) $$i^{2}=j^{2}=k^{2}=i\cdot j\cdot k=-1\;,\;i\cdot j=k\;,\;i\cdot j=-j\cdot i$$

the last relation in (34) shows the anti-commutativity of the quaternion basis and the role of the sign.

This attempt has to be put in perspective with the more well-known Boole’s method, used in the development of his logical calculus, where symbols are interpreted with the alphabet {0,1} when they obey the idempotence equation $x^{2}=x$, admitting only two possible solutions: 0 and 1 [5,12].

Boole concludes in his note [23]: “…*upon examination it will be found that these systems of interpretation are founded upon a principle of naming, as the one which I have proposed is founded upon a principle of operation. And I think it not foreign to the subject to remark, that the symbolical forms of common language as exhibited in the calculus of logic may indifferently be referred to the one or the other of these modes of conception*”.

So he discusses implicitly the duality between *naming* with a *sign* which represents *logical semantics* and *operation* which represents *logical syntax*.

The geometric properties are obvious when using quaternions. More specifically a unitary quaternion represents a rotation operation through an angle α around the axis n with spherical direction angles θ and φ. This gives the following expressions for the unitary quaternion coefficients named the *Euler–Rodrigues formula* (its importance is thoroughly discussed in [24]):

(35) w=cosα2 , x=sinα2sinθcosφ , y=sinα2sinθsinφ , z=sinα2cosθ

The coefficients in (35) lead to the composition rule for rotation operations asserting that any product of two rotations gives another rotation (*Euler’s rotation theorem*).

### 4.3. Pauli and Weyl–Heisenberg Operators and Their Semantic and Syntactic Interpretation in Logic

Rotation operators can also be expressed using the Pauli matrices ${\hat{\sigma }}_{x}$, ${\hat{\sigma }}_{y}$ and ${\hat{\sigma }}_{z}$. This can be understood because of the well-known isomorphism with the quaternion basis by: $i=-i{\hat{\sigma }}_{x}$, $j=-i{\hat{\sigma }}_{y}$ and $k=-i{\hat{\sigma }}_{z}$. The rotation operator can be derived directly from (33) and (35) and is expressed in the usual way in quantum mechanics as:

(36) $${\hat{R}}_{n\left(\theta ,\varphi \right)}\left(\alpha \right)=\exp-i({\hat{\sigma }}_{n}\alpha /2)\;\mathrm{with}\;{\hat{\sigma }}_{n}=\sin\theta \cos\varphi {\hat{\sigma }}_{x}+\sin\theta \sin\varphi {\hat{\sigma }}_{y}+\cos\theta {\hat{\sigma }}_{z}$$

Pauli matrices act on the 2-dimensional Hilbert space, their matrix representation is:

(37) $${\hat{\sigma }}_{x}=\left(\begin{array}{cc} 0 & 1\\ 1 & 0 \end{array}\right)\;,\;{\hat{\sigma }}_{y}=\left(\begin{array}{cc} 0 & -i\\ i & 0 \end{array}\right)\;,\;{\hat{\sigma }}_{z}=\left(\begin{array}{cc} 1 & 0\\ 0 & -1 \end{array}\right)$$

The principal relations are:

(38) $$\left\{{\hat{\sigma }}_{x},{\hat{\sigma }}_{z}\right\}={\hat{\sigma }}_{x}\cdot {\hat{\sigma }}_{z}+{\hat{\sigma }}_{z}\cdot {\hat{\sigma }}_{x}=0\;,\;{\hat{\sigma }}_{z}\cdot {\hat{\sigma }}_{x}=i{\hat{\sigma }}_{y}\;,\;{\hat{\sigma }}_{x}\cdot {\hat{\sigma }}_{y}\cdot {\hat{\sigma }}_{z}=iI$$

For an arbitrary direction n on the Bloch sphere, the Pauli matrix generalization ${\hat{\sigma }}_{n}$ is given in (36). The property of involution ${\hat{\sigma }}_{n}{}^{2}=I$ leads naturally to anti-commutativity ($\left\{{\hat{\sigma }}_{x},{\hat{\sigma }}_{z}\right\}=0$) because when squaring the operator in (36) all cross terms in the product must vanish to assure unity.

An interesting consequence of anti-commutativity is that the action of one of the Pauli matrices, for example ${\hat{\sigma }}_{x}$, on an eigenstate |+z〉 of one of the other Pauli matrices, for example ${\hat{\sigma }}_{z}$, generates the complementary eigenstate |−z〉. This can be shown using the eigenvalue equations and anti-commutativity:

(39) $${\hat{\sigma }}_{x}\cdot {\hat{\sigma }}_{z}\left|\pm z>\;=\left(\pm 1\right){\hat{\sigma }}_{x}\right|\pm z>\;=-{\hat{\sigma }}_{z}\cdot {\hat{\sigma }}_{x}|\pm z\rangle \;\mathrm{so}\;{\hat{\sigma }}_{z}\left({\hat{\sigma }}_{x}|\pm z\rangle \right)=\left(\mp 1\right)\left({\hat{\sigma }}_{x}|\pm z\rangle \right)$$

(40) $$\mathrm{giving}\;{\hat{\sigma }}_{x}|+z\rangle \;=|-z\rangle \;\;\mathrm{and}\;{\hat{\sigma }}_{x}|-z\rangle \;=|+z\rangle$$

The operation in (40) corresponds to logical binary negation which complements, for example, the state |+z〉 = |0〉  into |−z〉 = |1〉. By these arguments one observes that the basic logical operation of binary negation is a consequence of anti-commutativity.

The truth values of a logical operator represent its *logical semantics* and here in (39,40) the action of the logical operator on an input state represents its *logical syntax*. In this very simple example using the Pauli matrices as Eigenlogic operators, one has simultaneously a semantic representation by the eigenvalues of the diagonal Pauli matrix ${\hat{\sigma }}_{z}$ and a syntactic representation by a permutation operation represented by the Pauli matrix ${\hat{\sigma }}_{x}$.

When considering one qubit the only non-trivial logical operation is negation operated by the X gate. In quantum computation one must also consider logical operations on two and three qubits, these are also permutations as is the case for example for the CONTROL-NOT gate $C_{\mathrm{NOT}}$ (see the matrix form in (14)) or the Toffoli gate TO.

Most of the times operations are done on the computational basis which is the eigenbasis of ${\hat{\sigma }}_{z}$, this means that operators corresponding to quantum gates are represented in their non-diagonal syntactic form. The semantic diagonal form is rarely used except in some specific problems using physical Hamiltonians as is done in the field of *quantum simulation* for optimization and minimization problems seeking energies and ground states.

More developments are needed to give a complete semantic-syntactic picture, including, for example, 2 and 3 qubit gates. An interesting outlook discussing logical duality, in the context of quantum computing gates, was proposed recently in [25].

The quantum state generation process shown in (39) and (40) can be generalized for a d-dimensional multi-level system *(qudit*) by using the generalization of the Pauli matrices given by the operators of the Weyl-Heisenberg group $X_{d}$ and $Z_{d}$:

(41) $$X_{d}=\left(\begin{array}{c} \begin{array}{cc} 0 & 0\\ 1 & 0 \end{array}\;\begin{array}{cc} \cdots \;0 & 1\\ \cdots \;0 & 0 \end{array}\\ \begin{array}{cc} \vdots & \vdots \\ 0 & 0 \end{array}\;\begin{array}{cc} \ddots & \vdots \\ \cdots \;1 & 0 \end{array} \end{array}\right)\;\;,\;X_{d}|m\rangle \;=|m+1\rangle \;,\;X_{d}{}^{d}=I_{d}$$

and

(42) $$Z_{d}=\left(\begin{array}{c} \begin{array}{cc} 1 & 0\\ 0 & \omega_{d} \end{array}\;\begin{array}{cc} \cdots & 0\\ \cdots & 0 \end{array}\\ \begin{array}{cc} \vdots & \vdots \\ 0 & 0 \end{array}\;\begin{array}{cc} \ddots & \vdots \\ \cdots & \omega_{d}{}^{d-1} \end{array} \end{array}\right)\;\;,\;Z_{d}|m\rangle \;=\omega_{d}|m\rangle \;,\;\omega_{d}=e^{i\frac{2\pi }{d}}\;,\;Z_{d}{}^{d}=I_{d}$$

$X_{d}$ is usually named the *shift operator* and its representation is a *circulant matrix* and $Z_{d}$ is the *phase operator* and its representation is a diagonal matrix. The number $\omega_{d}$ is the d^th^ root of unity. These operators do not commute and obey a rule which can be considered as a generalization of the anti-commutation rule for Pauli matrices:

(43) $$Z_{d}\cdot X_{d}=\omega_{d}\;X_{d}\cdot Z_{d}$$

By the same procedure as the one given in (39) one can show that all states of a given eigenbasis of one operator are generated by the other operator. This is also clear by looking directly at the action of the shift operator $X_{d}$ on the state |m〉, which is an eigenstate of $Z_{d}$, giving the state |m+1〉, so by applying successively this operator one can generate all the other states of the basis.

Another interesting property is that the unitary transformation from $Z_{d}$ to $X_{d}$ is the *discrete Fourier transform* operator, named here $DFT_{d}$, having a *Vandermonde matrix* structure of basis $\omega_{d}$.

(44) $${\left(DFT_{d}\right)}_{ij}=\frac{1}{\sqrt{d}}\omega_{d}{}^{ij}\;,\;DFT_{d}{}^{4}=I_{d}\;,\;DFT_{d}{}^{-1}\cdot Z_{d}\cdot DFT_{d}=X_{d}$$

Considered as a quantum gate, this operator corresponds to a *Quantum Fourier Transform*.

The semantics is here represented by the eigenvalues of the reference Eigenlogic operator $Z_{d}$. The eigenvalues are the d^th^ roots of unity $\omega_{d}$, in particular, as described above, for a binary system the semantics is given by the square roots of unity: ±1.

The syntax on the other hand is represented by the shift operator $X_{d}$. The operator $X_{d}$ possesses the same eignevalues as $Z_{d}$ but is not the reference operator for the Eigenlogic system under considerartion.

The link to many-valued logic is straightforward: the *many-valued negation* introduced by Emil Post in [10] is exactly the shift operation m→m+1. So multi-level physical systems are directly linked to many-valued logic and an interesting guideline for future developments is that the Quantum Fourier Transform becomes a mediator between logical syntax and logical semantics.

The operators of the Weyl–Heisenberg group have been much investigated in relation to the problem of the so called MUBs (*Mutually Unbiased Bases*) [26]. Here the simplest case of Pauli matrices in dimension 2 gives 3 MUBs each being an eigenbasis of one of the three operators ${\hat{\sigma }}_{x}$, ${\hat{\sigma }}_{y}$ and ${\hat{\sigma }}_{z}$ (see (37)). The important property is that every basis vector from one MUB is uniformly distributed on all the basis vectors of the other MUBs. For example considering the eigenbasis vectors of ${\hat{\sigma }}_{x}$ and ${\hat{\sigma }}_{z}$ one has:

(45) $$|\langle \pm x|\pm z\rangle |=\frac{1}{\sqrt{2}}$$

The general rule being that d+1 MUBs exist when the dimension d of the Hilbert space is a prime number or a power of a prime number, this includes the n-qubit systems having dimensionality $d=2^{n}$ as thoroughly discussed in [27]. In the other cases, the number of MUBs is less than d+1 but one can always find at least 2 MUBs for every finite dimension. In particular there always exist two MUBs which are the two eigenbasis of the above discussed operators $X_{d}$ and $Z_{d}$ defined in (41,42).

As for the binary case more work is needed to scale up to higher arity for example in order to define universal many-valued logical operators such as the Min and Max operators obtained in Section 2.4 for the particular case of a balanced ternary logical system.

The links between logic and non-commutativity could also be considered for another very important topic in quantum information: *Quantum Error Correcting* (QEC) coding protocols. They permit actual quantum computers to overcome the problem of *decoherence* [15]. Popular QEC codes are the *stabilizer codes* where errors can be detected and corrected in a quantum circuit using a qubit *overhead* (increasing the number of qubits) with specially designed stabilizer operators. Briefly stated the eigenvalue −1 of a stabilizer operator corresponds to a detected error and the eigenvalue +1 to no error. Because of this eigenvalue structure these operators correspond to Eigenlogic involution operators G as discussed in Section 2.2. Recently the QEC code stabilizer formalism has also been applied to qutrit quantum circuits [28]. The detected errors correspond in this case to the phases $e^{i\frac{2\pi }{3}}$ and $e^{-i\frac{2\pi }{3}}$ which are two cubic roots of unity $\sqrt[{3}]{1}$. The other root is 1 and corresponds to no detected error. Also here one can make parallels with the qutrit Eigenlogic Weyl-Heisenberg group operators in (41) and (42) for dimension d=3.

## 5. Logical Conjectures on Universality, Predicate Logic, Normal Forms and Combinatory Logic

Here we will propose some conjectures and perspectives for Eigenlogic that could inspire a work-program for a quantum-like general consistent approach in logic using operators.

### 5.1. Truth Table Method and the Eigenlogic Interpretation of Universal Quantum Gates

Emil Post showed that syntax and semantics are linked by demonstrating the *consistency* and *completeness* of a finite logical system [10]. He also showed that truth tables, representing logical *semantics*, are axiomatic at the same level as the logical conjunctive and disjunctive canonical forms representing the logical *syntax.* This has an important consequence: logical universality can be shown using different methods, using syntax with the universal logical connectives but also using the semantics by directly inspecting the truth tables.

All logical operations are uniquely described by their truth table. On Table I all the truth tables are shown for the 16 binary logical connectives for two inputs (arity-2). In binary propositional logic 8 logical connectives when combined with the one input (arity-1) negation connective NOT form a universal logical set. These are AND, OR, NOR, NAND, implication A⇒B, non-implication A⇏B, inverse implication A⇐B and inverse non-implication A⇍B. The 8 remaining ones are non-universal, these are the logical projectors A and B and their negations ¬A and ¬B, equivalence ≡, exclusive disjunction XOR, contradiction F and tautology T.

Looking at the four values of the truth tables given in Table 1, one notices an interesting fact: the universal logical connectives have an unbalanced truth-table structure; more precisely, among the four truth-values the number of F (False) and T (True) are always odd whereas the non-universal connectives have always an even number of F and T. In Eigenlogic this fact can be transposed to the eigenvalue structure. For the involution logical operators with eigenvalues {+1,−1} the universal logic operators correspond then to *non-separable* (also called *non-local*) quantum gates, meaning that they cannot be expressed as a single Kronecker product [15,16].

As an example, let us take the Eigenlogic involution conjunction $G_{\mathrm{AND}}$ which is equivalent to the CONTROL-Z quantum gate $C_{Z}$ of Section 2.3. Considering its operator form in (11) and expressing it as a function of the seed operator Z it is clear that it cannot be put in the form of a single Kronecker product of two operators but is a sum of Kronecker products.

(46) $$G_{\mathrm{AND}}=C_{Z}=\frac{1}{2}\left(I_{4}+U+V-U\cdot V\right)=\frac{1}{2}\left(I_{2}⊗I_{2}+Z⊗I_{2}+I_{2}⊗Z-Z⊗Z\right)$$

The other universal logic operators, as clearly shown on Table 1, have a similar operator structure as in (46). On the other hand the remaining 8 non-universal logic operators are Kronecker products, for example:

(47) $$G_{A}=U=Z⊗I_{2}\;,\;G_{B}=V=I_{2}⊗Z\;,\;G_{\mathrm{XOR}}=Z⊗Z$$

From a quantum computing perspective non-local operators are essential for building universal quantum gates. The CONTROL-NOT gate $C_{\mathrm{NOT}}$ (see Section 2.3), which is a non-local 2-qubit gate, associated with a non-trivial one-qubit gate (essentially a rotation operator) constitutes a universal quantum gate set. It is also important to notice that only these non-local control gates have an entangling power when applied on qubits that are not eigenstates [15,16]. This states clearly the correspondence between universal quantum logic gates and entanglement which is an accepted fact in quantum computing.

### 5.2. Towards First Order Eigenlogic: A Link with Grover’s Algorithm

Using two maximally incompatible logical families with logical eigensystems associated to the X and Z gates (*resp.* the ${\hat{\sigma }}_{x}$ and ${\hat{\sigma }}_{z}$ Pauli operators) one gets an interesting outlook: the quantum Grover amplification gate [15], used in the *Grover algorithm* [29], corresponds to the multi-qubit involution Eigenlogic negated disjunction operator NOR in the ***X*** system. This operator can be interpreted in the Z system as a predicative logical *existential connective* ∃.

**Figure 1:**
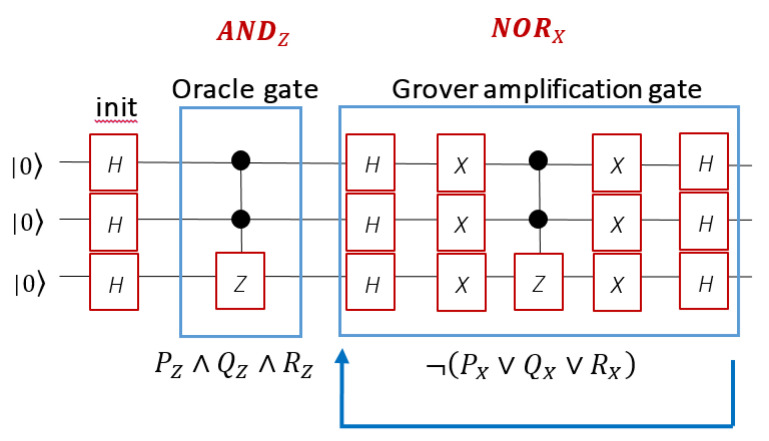
Quantum circuit for the phase oracle followed by the Grover amplification gate.

An example of a circuit implementing the Grover algorithm on 3 qubits with a phase oracle gate followed by the Grover gate is shown in Figure 1. The Grover algorithm looks for one element among 8, here the state |111〉, that satisfies the logical propositional clause P represented by the phase oracle gate. The phase oracle is here a doubly-CONTROL-Z gate, $CC_{z}$, which corresponds to a 3-qubit Eigenlogic conjunction (AND, ∧). The Grover amplification gate is the 3-qubit Eigenlogic NOR diagonal in the X system eigenbasis. Restated in the language of first order logic this circuit operates the following logical proposition:

(48) $$\exists a\;P\left(a\right)\;\equiv \;\neg \left(P_{X}\vee Q_{X}\vee R_{X}\right)\;\left[P_{Z}\wedge Q_{Z}\wedge R_{Z}\right]$$

This argument derives from the *Skolemization* methods that provide constructive derivability approaches in first-order predicate logic using propositional logic.

The predicate proposition ∃a P(a) (“there exists a satisfying P(a)”) can be decomposed, when considering a finite domain of variables, using a succession of disjunction and conjunction connectives. This fact is well known in logic.

This is just a first step in the direction of generalizing Eigenlogic to first order logic but we think that it could lead to new insights, for example, in the context of well-established quantum algorithms.

### 5.3. The Production Systems of Emil Post and the Role of Non-Commutativity

Emil Post proved that any formal system (e.g., any Turing machine) can be put into different reduced forms (the *Post production systems*) [30] and in particular in the canonical *normal form*, defined by one single axiom, giving a production rule for *word-strings*:

(49) L A produces A R

In the normal form (49) A is the argument string, *L* and R are the production strings, L A is the input string and A R the output string. So any string beginning with L may be replaced by the string in which L is removed and R is attached at the other end.

This formalism has been recently considered using matrices, where words in strings are replaced by an alphabet of elementary 2×2 matrices [31].

Using the non-commutativity of operators, the formulation (49) can also be understood as the action of non-commuting operators. Let us express this by the *commutators* B and D defined as:

(50) B=[L,A]=L⋅A−A⋅L , D=[R,A]=R⋅A−A⋅R with L⋅A=A⋅R

this gives the following relation:

(51) $$B=A\cdot \left(R-L\right)\;,\;D=\left(R-L\right)\cdot A\;\mathrm{giving}\;D\cdot A^{-1}=A^{-1}\cdot B$$

The last expression in (51) can be considered as the operator analog of the normal form (49) using reversible operators. The case of commutation corresponds to R=L giving B=D=0 which signifies that the system cannot produce any new output, the output equals the input and so there is no change in the system.

A link with quantum computation could be established using these logical production systems using non-commuting reversible quantum gates.

### 5.4. Qauntum-Like Combinatory Logic

In 1924, Moses Schönfinkel [32] introduced a method in logic named *Combinatory Logic*. This research was part of the Hilbert program which aimed to formulate all the fields of mathematics in a consistent logic system by means of a finite set of axioms and inference rules. Haskell Curry successively improved and completed the research on combinatory logic [33]. This led to the development of *functional programming languages* such as *Haskell*, and *Erlang*.

Combinatory logic uses abstract operators (called *combinators*) to compose and to transform operators and arguments. The combinators I, K, S, B and C have the following rules:

(52) Ix=x ; Kxy=x ; Sxyz=xz(yz) ; Cxyz=xzy,

where the symbols x, y and z denote the position on which the combinator acts and can designate numbers, functions or operators. Brackets are used to denote the order of the application and are omitted when the sense is clear. The two combinators S and K form a basis for all combinatory logic, shown by the following relations:

(53) I=SKK ; B=S(KS)K ; C=S(BBS)(KK)

Schönfinkel’s method permits to translate first order logic *well-formed formulas* into expressions without variables using only the combinators I, K, S, B and C. In this way, combinatory logic solves the decision problem, in a first-order logic proposition, of how logical variables are bounded to the universal quantifier ∀ and the existential quantifier ∃ without the need of any variable,

Can this formulation be converted using the operators discussed in this paper or more generally using quantum gates? Research has been undertaken proposing a reversible version of combinatory logic in [34] where the motivation was the development of a form of semantics based on linear operator algebras supporting a compositional approach to (probabilistic) program analysis. The author of the paper, Alessandra di Pierro, considers that “…*reversible combinatory logic can in principle be used for a (maybe highly inefficient) translation of classical into quantum computation*”.

A tentative approach to link combinatory logic to quantum computation could consist in identifying the different operations of substitution, elimination, permutation, etc., with equivalent operations obtained using common quantum gates. There are some nice tricks in quantum computation that could be used to fulfill these requirements. These tricks use two-qubit quantum gates. For example using the non-local CONTRIOL-Z gate $C_{Z}$ (see Section 2.3) one obtains the function of elimination needed for the combinator K, the trick is [15]:

(54) $$C_{Z}\;\cdot \;\left(X⊗Z\right)\;\cdot \;C_{Z}=X⊗I_{2}$$

In (54) X⊗Z represents the Kronecker product ⊗ of the Pauli operators $X={\hat{\sigma }}_{x}$ and $Z={\hat{\sigma }}_{z}$, the dot ⋅ corresponds to matrix product. One sees that Z appearing on the *l.h.s.* in (54) has been eliminated. The interesting property here is also reversibility because $C_{Z}\;$ is an involution operator, $C_{Z}{}^{2}=I_{4}$, so relation (54) can be reversed.

Also, the involution SWAP gate [15] $U_{swap}$ could be used for permutation, by the known relation:

(55) $$U_{swap}\;\cdot \;\left(P⊗Q\;\right)\cdot \;U_{swap}\;=Q⊗P$$

which could be related to the combinator C.

Of course this is only a tentative approach and needs to be finalized, but there seems to be no technical reason why these combinatory operations could not be realized with quantum gates.

## 6. Conclusions

This work shows a new *quantum-like* approach in logic. It stems from recent research having contributed to the *Quantum Interaction* community, where quantum formalism is used outside the field of physics. Research efforts in this community have, for example, presented global quantum-like approaches for humanities [35]. The approach presented in this paper is also related to quantum-like models that have been used for studies in *Information Retrieval* [36] and *Semiotics* [37].

Linear algebra is used nowadays everywhere because it has become the standard tool of the computer based disciplines related to the *Big Data* revolution such as *Machine Learning*, *Neural Networks*, and *Artificial Intelligence* etc. Formalizing logic in an operator matrix language, as is done in Eigenlogic, could bring benefits because logic can be treated directly in this framework. This new approach using logic and operators could be inspired by recently proposed quantum-like machine learning methods using density matrix operators [38].

We also pointed out in this research the central role played by truth-table logical semantics as a major tool for adapting logic to physics showing the importance of the choice of the logical alphabet. Eigenlogic treats semantics in a most natural way because it is essentially given by the eigenstructure of the logic operators. Also the much investigated syntactic-semantic duality in logic is in some way unified in this approach.

History shows that, at their foundations, logic and quantum mechanics travelled on the same roads, and there were many interactions between mathematicians and physicists. Inspired by several approaches in logic, we made some conjectures that could *in fine* bring to a consistent logical program using the common tools which are being developed for the development of the quantum computer such as quantum gates and quantum algorithms. We aimed to open some paths in this direction.

## Figures and Tables

**Table 1:** Sixteen two-argument (arity2) truth-tables and logic operators for {0,1} and {+1,−1}.

Connective A, B	Truth Table {F,T} ,	Projection {0=F,1=T} ,	Involution{+1=F,−1=T}
False ; F ; ⊥	F F F F	0	+I
NOR	T F F F	I−A−B+A⋅B	(1/2) (+I −U −V −U⋅V)
A ⇍ B	F T F F	B−A⋅B	(1/2) (+I−U+V+U⋅V)
¬A	T T F F	I−A	−U
A ⇏ B	F F T F	A−A⋅B	(1/2) (+I+U−V+U⋅V)
¬B	T F T F	I−B	−V
XOR ; A⊕B	F T T F	A+B−2A⋅B	U⋅V=Z⊗Z
NAND ; A↑B	T T T F	I−A⋅B	(1/2) (−I−U−V+U⋅V)
AND ; A∧B	F F F T	A⋅B=Π⊗Π	(1/2) (+I+U+V−U⋅V)
A≡B	T F F T	I−A−B+2A⋅B	−U⋅V
B	F T F T.	B=I⊗Π	V= I⊗Z
A⇒B	T T F T	I−A+A⋅B	(1/2) (−I−U+V−U⋅V)
A	F F T T	A=Π⊗I	U = Z⊗ I
A ⇐ B	T F T T	I−B+A⋅B	(1/2) (−I+U−V−U⋅V)
OR ; A∨B	F T T T	A+B−A⋅B	(1/2) (−I+U+V+U⋅V)
True ; T	T T T T	I	−I
